# Prognostic nomogram for multiple myeloma early relapse after autologous stem cell transplant in the novel agent era

**DOI:** 10.1002/cam4.5630

**Published:** 2023-04-06

**Authors:** Huixing Zhou, Yuan Jian, Juan Du, Junru Liu, Zhiyao Zhang, Guangzhong Yang, Guorong Wang, Ying Tian, Yanchen Li, Yin Wu, Wenming Chen, Weijun Fu, Juan Li, Wen Gao

**Affiliations:** ^1^ Department of Hematology Myeloma Research Center of Beijing, Beijing Chao‐Yang Hospital, Capital Medical University Beijing Chaoyang China; ^2^ Department of Hematology The Myeloma & Lymphoma Center, The Second Military Medical University Shanghai China; ^3^ Department of Hematology The First Affiliated Hospital to Sun Yat‐sen University Guangdong Guangzhou China; ^4^ Department of Hematology Shanghai Fourth People's Hospital, School of Medicine, Tongji University Shanghai China

**Keywords:** autologous stem cell transplantation, early relapse, multiple myeloma, nomogram

## Abstract

**Background:**

The present study intended to establish a predictive nomogram for early relapse (ER) (<12 months) after autologous stem cell transplantation (ASCT) in the novel drug era for multiple myeloma (MM).

**Patients and Methods:**

The nomogram was designed and constructed to a retrospective clinical data of newly diagnosed MM patients received novel agent induction therapy and subsequent ASCT at three centers in China from July 2007 to December 2018. The retrospective study was conducted among 294 patients in the training cohort and 126 in the validation cohort. The nomogram's predictive accuracy was evaluated by the concordance index, calibration curve and decision clinical curve.

**Results:**

The study cohort included 420 newly diagnosed MM patients, and 100 (23.8%) were identified as having ER, including 74 in the training cohort and 26 in the validation cohort. According to the result of multivariate regression in the training cohort, the prognostic variables included in the nomogram were high‐risk cytogenetics, LDH > UNL, and response less than very good partial response (VGPR) after ASCT. The calibration curve showed good fitness between the nomogram predictions and the actual observations and the nomogram was further validated by a clinical decision curve. The nomogram's C‐index achieved 0.75 (95% CI, 0.70–0.80), which was higher than that of the Revised International Staging System (R‐ISS) (0.62), ISS (0.59), and Durie–Salmon (DS) staging system (0.52). The discrimination ability of the nomogram in the validation cohort was superior to that of the other staging systems (C‐index: 0.73 vs. R‐ISS (0.54), ISS (0.55), and DS staging system (0.53)). DCA showed the prediction nomogram adds much more clinical utility. Different scores of the nomogram draw a distinction of OS.

**Conclusion:**

The present nomogram could serve as a feasible and accurate prediction of ER in novel drug induction transplantation‐eligible MM patients, which could help modify the post‐ASCT strategy for patients at high risk of ER.

## Graphical abstract

Early relapse post‐ASCT in MM makes patients suffer adverse outcomes, which is so far unpredictable. This study retrospectively enrolled data of transplant‐eligible MM patients from multicenters in China and established a predictive nomogram for ER. The C‐index demonstrates good predictive accuracy for survival and the calibration curve for the probability of survival showed good agreement between the prediction by the nomogram and actual observations, which could be used to modify the post‐ASCT strategy for those patients.

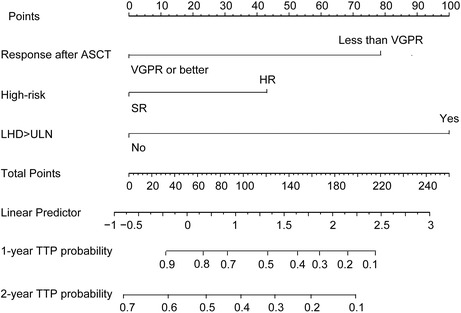

## 1 INTRODUCTION

The strategy of novel agent‐based induction therapy and high‐dose chemotherapy with followed ASCT has significantly prolonged the survival of MM patients in recent decades.^1^, ^2^, ^3^, ^4^, ^5^ Despite the exposure to novel agents and intensive ASCT consolidation, early relapse (ER) still represents an aggressive disease course and poor outcome. Almost 20% of patients relapse within 1 year after ASCT.^6^ These patients have a disease course characterized by early relapse (ER) and progression and suffer shorter survival.

Previous reports have emphasized that patients who experience ER within 1 or 2 years tend to suffer inferior survival despite the deep response to ASCT and several markers have been shown to provide information about the risk of ER.^7^, ^8^, ^9^ The study by Kumar et al.^10^ first reported ER as an independent adverse prognostic factor. The study reported 494 patients who received ASCT after conventional chemotherapy, showing that a high plasma cell labeling index at transplant, more than one line of treatment before ASCT, and response less than CR were predictive of relapse within 12 months. In the novel drug era, even though MM patient outcomes continue to improve with the introduction of novel drugs, ER continues to be a defining biological feature in MM.^11^, ^12^ Available evidence showed that the Revised International Staging System (R‐ISS) was predictive of ER (<24 months) after ASCT.^13^ Data from a group in Singapore^9^ showed that a suboptimal posttransplant response and ER are more critical than pretransplant factors in terms of survival impact. However, data from Chinese myeloma patients were limited and clinical and prognostic profiles of Chinese MM patients experiencing ER after ASCT would contribute to understanding disease characteristics to provide basis for making countermeasures and improving outcomes.

Considering the poor outcomes of ER, it is essential to recognize ER patients who are likely to experience a poor outcome with standard therapeutic approaches. As next‐generation novel drugs with multiple mechanisms of action have already sprung up, a delicate and personalized strategy of novel drugs could help improve the poor outcome of ER patients. To figure out the profile of Chinese ER patients in MM, we analyzed the clinical records of patients who received novel agents involved induction followed by ASCT from three Chinese centers. The objective of this study was to construct an effective prognostic nomogram for ER (<12 months) after ASCT in MM in the novel drug era that reflects important clinical features and can be used to guide real‐world clinical practice.

## 2 PATIENTS AND METHODS

### 2.1 Patients

This retrospective study was designed and performed on a cohort of newly diagnosed MM patients who underwent 4–7 cycles of novel agent‐based induction followed by ASCT from July 2007 to December 2018 at Beijing Chao‐Yang Hospital, Guangzhou Zhongshan Hospital, and Shanghai Changzheng Hospital. The exclusion criteria were as follows: salvage ASCT, secondary transplants, allogeneic stem cell transplant, unknown induction treatment, unknown relapse status, and traditional chemotherapy introduction. All patients provided informed consent for the use of their medical records. The Ethics Committee of Beijing Chao‐Yang Hospital approved this study following the Declaration of Helsinki.

### 2.2 Diagnosis and treatment

Both MM diagnosis and response were evaluated under the International Myeloma Working Group criteria.^14^ All patients received novel agent‐based (proteasome inhibitor or IMiDs) induction therapy followed by ASCT, including 84.6% bortezomib‐based regimens and 15.4% bortezomib–lenalidomide‐based regimens. Baseline FISH analysis was conducted after the enrichment of CD38 immunomagnetic beads targeting the following cytogenetic abnormalities: 1q21, 17p13.1, *t* (11;14), *t* (14;16), and *t* (4;14). Two hundred interphase nuclei were analyzed for each sample. The cutoff values were as follows: 10% for *t* (4;14), *t* (11;14), and *t* (14;16) and 20% for del(17p) and 1q21 gains.^11^

### 2.3 Follow‐up and ER

The response after ASCT was assessed at 80–120 days after ASCT. The ER group was defined as patients who progressed or relapsed within 12 months after ASCT. Time to progression (TTP) was defined as the time from ASCT to progressive disease or death. Overall survival (OS) was defined as the time from the first date of stem cell infusion to death or last follow‐up. In addition, patients who had undergone salvage ASCT and died within 12 months without evidence of myeloma‐related death were excluded. The non‐ER group was defined as patients who did not have disease progression or myeloma‐related death within 12 months after ASCT.

### 2.4 Statistics

Methods of Statistics to determine risk variables were executed by SPSS 22.0. The characteristics of patients were described using the median value for continuous variates and the frequency for categorical ones. A two‐sided Fisher exact test and Student's *t*‐test were used to calculate differences between groups for categorical and continuous variables, respectively. Kaplan–Meier Curve and the two‐tailed log‐rank test were used to analyze survival data using SPSS 22.0 and GraphPad Prism 7. Univariate analysis and multivariate analysis were conducted to compare variates among groups over time. Cox regression analysis was to investigate the validity of multivariate analysis for survival data. The multivariable logistic analysis was conducted to identify independent factors predicting ER.

A nomogram was calculated based on the filtered variables of multivariate analysis by the rms^15^ package in R version 4.1.2 (http://www.r‐project.org/). The process of randomization for patients into the training and validation cohorts was conducted using the formula in R. A final model selection was calculated by a backward step‐down selection process with criteria previously reported.^16^ The nomogram's performance was evaluated by the concordance index (C‐index) and the nomogram‐predicted probability was compared with observed Kaplan–Meier estimates of survival probability. Bootstraps with 1000 resamples were applied to this analysis. Comparisons between the different prognostic systems were performed with the rcorrp.cens package in Hmisc28^17^ in R. The prognostic prediction was more accurate with the larger C‐index. Validation of the prognostic nomogram was conducted in the validation cohort. The proportional hazard assumption was tested by “cox.zph” functions in R. Clinical utility for COX regression was estimated by decision curve analysis (DCA) using the “stdca.R" via Memorial Sloan Kettering Cancer Center [https://www.mskcc.org/departments/epidemiology‐biostatistics/biostatistics/decision‐curve‐analysis] in R which was modified based on “rmda” package in R.^18^ *p* < 0.05 indicated significance.

## 3 RESULTS

### 3.1 Patient demographics

Total of 420 newly diagnosed MM patients who received novel agents‐involved induction and subsequent ASCT were entered into this study. The study cohort was randomly separated into the training cohort (*n* = 297; 70%) and the validation cohort (*n* = 123; 30%). Median age of the total cohort was 53 years (range, 25–69 years). The median time from diagnosis to ASCT was 6.70 months. The median follow‐up time was 54 months. The patients were separated into ER/non‐ER groups based on the above definition. The study cohort included 420 newly diagnosed MM patients, and 100 (23.8%) were identified as having ER. The training cohort contained 74 (24.9%) and the validation cohort contained 26 (21.1%). The baseline demographics are detailed in Table 1.

**TABLE 1:** Patient characteristics of the early relapse group (ER) and non‐ER group.

		Training cohort (*n* = 297)					Validation cohort (*n* = 123)				
		ER group *N* = 74		Non‐ER group *N* = 223		*p*‐Value	ER group *N* = 74		Non‐ER group *N* = 223		*p*‐Value
Sex *n* (%)	Male	38	(51.4)	133	(59.6)	0.21	19	(73.1)	62	(63.9)	0.38
Median age		54 (25–67 years)		53 (25–69 years)			55 (43–64 years)		52 (30–67 years)		0.58
Heavy chain *n* (%)	IgG	35	(64.8)	122	(68.2)	0.69	15	(65.2)	48	(58.5)	0.30
	IgA	14	(25.9)	46	(25.7)		5	(21.7)	28	(34.1)	
	IgD	3	(5.6)	7	(3.9)		3	(13.0)	4	(4.9)	
	Non‐secretor	2	(3.7)	4	(2.2)		‐‐	‐‐	2	(2.4)	
Light chain *n* (%)	Kappa	37	(50.0)	128	(57.5)		14	(53.8)	50	(52.1)	0.55
	Lambda	35	(47.3)	93	(41.9)		12	(46.2)	44	(45.8)	
Renal function *n* (%)	A	42	(79.2)	137	(78.3)	0.52	11	(61.1)	50	(80.6)	0.09
	B	11	(20.8)	38	(21.7)		7	(38.9)	12	(19.4)	
LDH (U/L)^a^		189 (145, 272)		157 (125, 199)		0.003	187 (138, 271)		154 (128, 197)		0.11
Hemoglobin (g/L)^a^		98.5 (77, 112)		95 (78, 114)		0.96	88 (74, 104)		101 (88, 124)		0.15
BMPC (%)^a^		34.5 (19.0, 54.0)		33.5 (17.0, 49.5)		0.40	25.7 (14.0, 52.0)		27.3 (15.0,46.0)		0.91
β2‐microglobulin (mg/L)^a^		4.2 (2.4, 5.8)		3.3 (2.1, 5.8)		0.12	4.0 (2.9, 6.8)		2.8 (2.1, 4.8)		0.62
Calcium (mmol/L)^a^		2.4 (2.2, 2.6)		2.4 (2.3, 2.6)		0.75	2.7 (2.4, 2.9)		2.4 (2.2, 2.6)		0.13
High‐risk *n* (%)^*^		31	(41.9)	59	(26.5)	0.012	11	(42.3)	22	(22.7)	0.045
DS stage *n* (%)	I + II	11	(15.3)	29	(13.1)	0.71	4	(15.3)	12	(22.5)	0.15
	III	61	(84.7)	193	(86.9)		22	(84.6)	84	(87.5)	
ISS *n* (%)^*^	I	12	(16.2)	60	(26.9)	0.17	5	(19.2)	38	(39.2)	0.042
	II	29	(39.2)	79	(35.4)		8	(30.8)	34	(35.1)	
	III	33	(44.6)	84	(37.7)		13	(50.9)	25	(25.8)	
R‐ISS *n* (%)^*^	I + II	57	(77.0)	193	(76.5)	0.003	19	(73.1)	88	(90.8)	0.041
	III	17	(23.0)	30	(13.5)		7	(26.9)	9	(9.3)	

*Note*: High‐risk cytogenetics was defined as *t* (4;14), del17p or *t* (14;16).

Abbreviations: BMPC, bone marrow plasma cell; DS, Durie Salmon stage; LDH, lactate dehydrogenase; R‐ISS, revised international staging system.

^a^ Described as the median and quartile.

* *p* < 0.05.

In the training cohort, the rate of stage R‐ISS III group was more in ER than in non‐ER (23.0% vs. 13.5%, *p* = 0.003), and the ER group patients had a higher median lactate dehydrogenase (LDH) level (189 (145, 272) vs. 157 (125, 199), *p* = 0.003) and a higher rate of high‐risk cytogenetics (41.9% vs. 26.5%, *p* = 0.012).

### 3.2 Response and cytogenetics

The therapeutic response of the training cohort is detailed in Table 2. There were 66.8% of patients achieved VGPR, and this rate was higher in the non‐ER group than in the ER group (54.1%) (*p* = 0.048). At 3 month after ASCT, the rate of a VGPR or better response was higher in the non‐ER group (77.1% vs. 60.9%, *p* = 0.006). The complete response (CR) rate after ASCT in the non‐ER patients was higher than those in the ER group (52.9% vs. 39.2%, *p* = 0.041).

**TABLE 2:** The response after ASCT of the training cohort.

Response	ER group (*n* = 74)		Non‐ER group (*n* = 223)	
	Pre‐ASCT (*n*, %)	After‐ASCT (*n*, %)	Pre‐ASCT (*n*, %)	After‐ASCT (*n*, %)
CR	21 (28.4)	29 (39.2)	64 (28.7)	118 (52.9)
VGPR	19 (25.7)	16 (21.6)	85 (38.1)	54 (24.2)
VGPR and better	40 (54.1)	45 (60.9)	149 (66.8)	172 (77.1)
PR and less	34 (45.9)	29 (39.2)	74 (33.2)	51 (22.9)

Abbreviations: CR, complete response; PR, partial response; VGPR, very good partial response.

Cytogenetic profiles based on FISH are listed in Table 3. There were more patients with high‐risk cytogenetics (del17p, *t* (4;14), *t* (14;16)) in the ER group (41.9% vs. 25.6%, *p* = 0.02). Del17p and *t* (4;14) were more prevalent in the ER group (20.3% vs. 11.2%, *p* = 0.038; 20.3% vs. 4.9%, *p* = 0.038, respectively).

**TABLE 3:** Cytogenetic profiles based on interphase fluorescence in situ hybridization

Abnormal cytogenetics	ER (*n* = 74)	Non‐ER (*n* = 223)
1q21 gain *n* (%)	36 (48.6)	96 (43.0)
Del17p *n* (%)*	15 (20.3)	25 (11.2)
t(4;14) *n* (%)*	15 (20.3)	11 (4.9)
t(11;14) *n* (%)	7 (9.5)	31 (13.9)
t(14;16) *n* (%)	5 (6.8)	5 (2.2)
High‐risk *n* (%)^a^ ^,^ *	31 (41.9)	59 (26.5)

^a^ High risk was defined as del17p, *t*(4;14) or *t*(14;16).

* *p* < 0.05.

### 3.3 Survival of the training cohort

**FIGURE 1:**
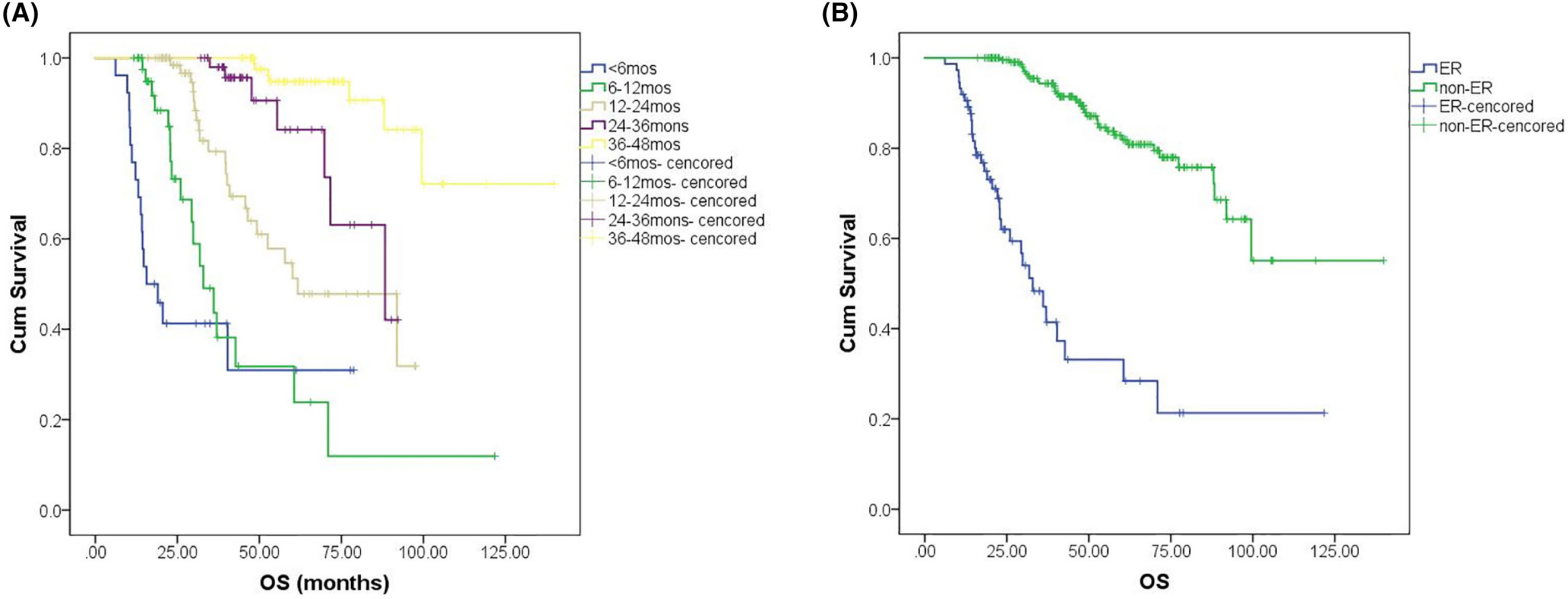
Median OS was prolonged with increasing TTP (A). Median OS in the ER and non‐ER groups (B).

The median TTP was 39.8 months (95% CI: 32.5–70.6), and the median OS was 41.7 months (95% CI: 14.36–93.8) in the training cohort. The Kaplan–Meier Curve showed that with the rise in the median TTP [<6 m (*n* = 38), 6–12 m (*n* = 62), 12–24 m (*n* = 119), 24–36 m (*n* = 83) and > 36 m (*n* = 118)], the median OS was significantly prolonged (<6 m: 15.5 m, 95% CI: 8.3–22.8; 6–12 m: 32.9 m, 95% CI: 24.5–41.4; 12–24 m: 61.7 m, 95% CI: 36.8–86.6; 24–36 m: 88.3 m, 95% CI: 57.8–118.9; >36 m: not reached, *p* < 0.001) (Figure 1A). The median OS was statistically shorter in the ER group than that in the non‐ER group (32.9 months (95% CI: 24.6–41.2) vs. not reached, *p* < 0.001) (Figure 1B).

### 3.4 ER was an adverse‐independent predictor in the training cohort

Kaplan–Meier analysis confirmed that OS was statistically shorter in the ER group than in the non‐ER group (Figure 1B). Regression analysis was conducted to determine potential prognostic factors for OS, including ISS stage, R‐ISS stage, less than VGPR after ASCT, LDH > ULN, R‐ISS III stage, and ER. Multivariate analysis showed that ER (HR 8.09, 95% CI: 4.35–15.0, *p* < 0.0001) and high‐risk cytogenetics (HR 2.31, 95% CI: 1.18–4.51, *p* = 0.015) were independent adverse factors for OS (Table 4).

**TABLE 4:** Univariate and multivariate analysis of overall survival.

	Univariate analysis			Multivariate analysis		
	*p*‐Value	HR	95% CI	*p*‐Value	Adjusted HR	95% CI
Early relapse	<0.0001	8.47	5.20–13.79	<0.0001	8.09	4.35–15.0
ISS III	0.002	2.9	1.41–6.02	0.11	2.18	0.85–5.64
High‐risk cytogenetics	<0.0001	2.61	1.63–4.19	0.015	2.31	1.18–4.51
Less than VGPR after ASCT	0.016	1.66	1.02–3.01	0.02	2.31	1.18–4.51
LDH > ULN	0.003	2.40	1.35–4.27	0.43	1.32	0.66–2.64

Abbreviations: ASCT, autologous hematopoietic stem cell transplantation; CI, confidence interval; ER, early relapse; HR, hazard ratio; LDH, lactate dehydrogenase; R‐ISS, Revised International Staging System; VGPR, very good partial response.

### 3.5 Univariate analysis of ER in the training cohort

The median follow‐up time was 45 months (10–139 months). The median TTP and OS were 29.8 months (95% CI: 10.5–87.6) and 40.5 months (95% CI: 7.7–115.9), respectively. The details of the univariate analysis of the training cohort are listed in Table 5. Variables in the univariate analysis included DS stage, renal function, ISS stage, high‐risk cytogenetics, R‐ISS stage, LDH group (LDH > ULN vs. standard), bone marrow plasma cell percentage (BMPC) ≥ 60%, response before ASCT (less than VGPR vs. VGPR or better), and response after ASCT (less than VGPR vs. VGPR or better).

**TABLE 5:** Univariate analysis of ER in the training cohort.

Variable	ß	SE	Wald χ^2^	HR	95% CI	*p*‐value
ISS stage (III vs. I + II)	0.243	0.234	1.077	1.28	0.81–2.02	0.299
DS stage (III vs. I + II)	0.066	0.357	0.034	1.07	0.53–2.15	0.853
Renal function (B vs. A)	−0.71	0.339	0.044	0.93	0.48–1.81	0.834
High‐risk cytogenetics	0.651	0.230	8.003	1.92	1.22–3.01	0.005
Response before ASCT	0.106	0.255	0.174	1.11	0.67–1.83	0.677
R‐ISS stage (III vs. I + II)	0.664	0.264	6.328	1.94	1.16–3.26	0.012
Response after ASCT	1.805	0.238	20.719	2.96	1.86–4.72	<0.0001
LDH > ULN	1.277	0.231	30.499	3.58	2.28–5.64	<0.0001
BMPC≥60%	0.138	0.289	0.228	1.15	0.65–2.02	0.633

Abbreviations: ASCT, autologous stem cell transplantation; BMPC, bone marrow plasma cells; CI, confidence interval; DS stage, Durie‐Salmon staging system; ER, early relapse; HR, standard error; ISS, International Staging System; LDH, lactate dehydrogenase; Renal function A, relatively normal renal function (serum creatinine value) < 2.0 mg/dL or serum creatinine <177umol/L; Renal function B, abnormal renal function (serum creatinine value) ≥ 2.0 mg/dL or serum creatinine≥177umol/L; SE, standard error; ULN, upper limit of normal.

### 3.6 Multivariate analysis of ER in the training cohort

Multivariate analysis of ER was then performed with binary logistic regression. Based on univariate analysis, high risk, response after ASCT, LDH > ULN, and R‐ISS stage III were considered in the multivariate analysis. However, the R‐ISS stage is evaluated according to ISS stage, LDH level, and high‐risk cytogenetics, so R‐ISS was not incorporated in the model to avoid confounding factors. The multivariate analyses demonstrated that high‐risk cytogenetics, response after ASCT (less than VGPR), and LDH > ULN were independent risk factors for ER (Table 6).

**TABLE 6:** Multivariate analysis of ER in the training cohort.

Variable	ß	SE	Wald *χ* ^2^	OR	95% CI	*p*‐Value
High‐risk cytogenetics	−0.777	0.264	8.633	0.46	0.09–0.28	0.003
Response after ASCT	−1.870	0.297	39.648	0.15	0.09–0.28	<0.0001
LDH > ULN	−1.915	0.288	44.299	0.15	0.08–0.26	<0.0001

Abbreviations: ASCT, autologous stem cell transplantation; CI, confidence interval; ER, early relapse; LDH, lactate dehydrogenase; OR, odd ratio; SE, standard error; ULN, upper limit of normal.

### 3.7 Prognostic nomogram for ER in the training cohort

**FIGURE 2:**
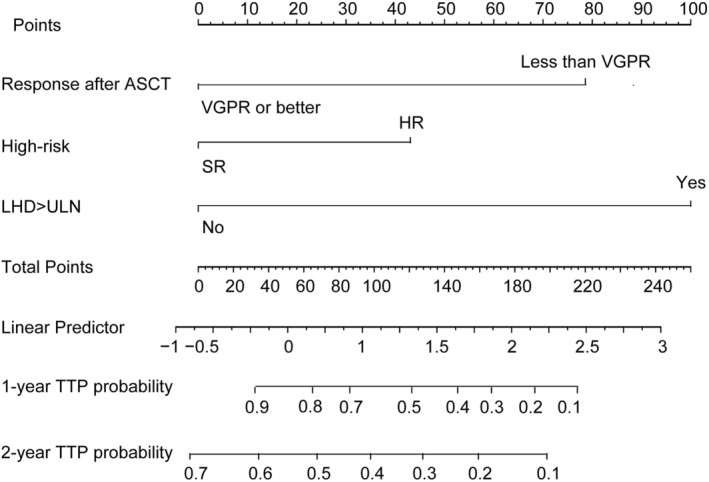
Nomogram for predicting ER after ASCT. To calculate survival probability, each predictor is located on the axis with a corresponding score on the points axis. Each point is added to the total points axis, and the total points represented the probability of 1‐ or 2‐year TTP.

**FIGURE 3:**
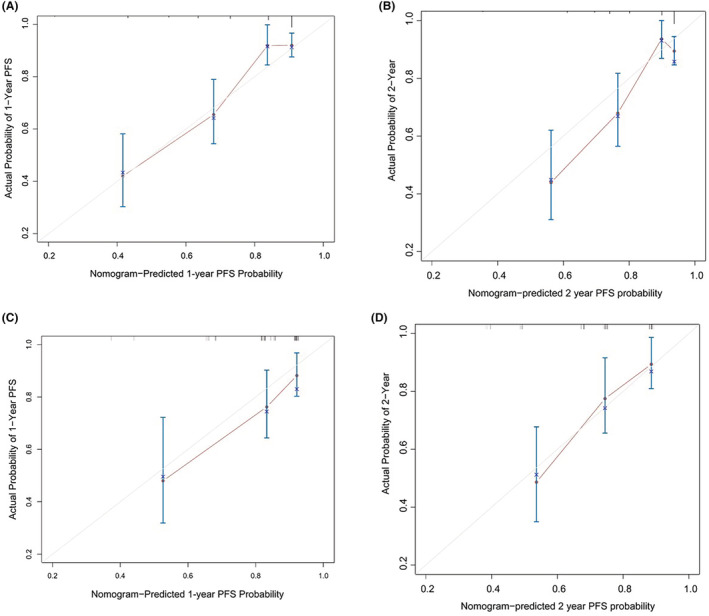
The calibration plot curve for survival prediction in the training cohort at (A) 12 months and (B) 24 months and in the validation cohort at (C) 12 months and (D) 24 months. The *x*‐axis listed the nomogram predicted probability of ER and the *y*‐axis showed the actual survival probability.

The predictive nomogram model that incorporated all of the independent variates for ER in the training cohort is presented in Figure 2. The C‐index of 0.75 (95% CI, 0.70–0.80) was achieved for ER prediction. The calibration plot was showed well agreement in 12 months and 24 months prediction after ASCT between the prediction and actual probabilities (Figure 3A, B).

**FIGURE 4:**
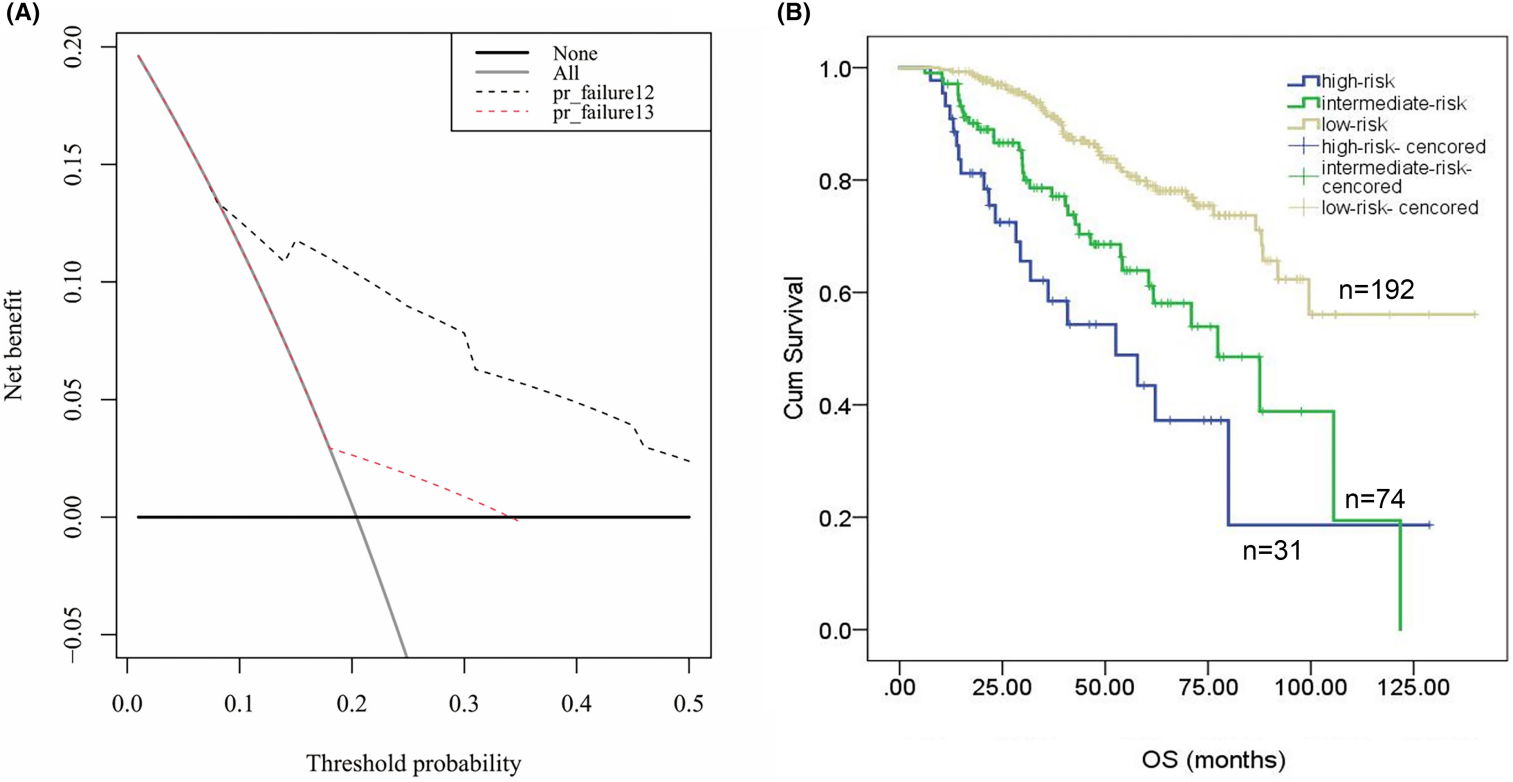
Decision curve analysis for the nomogram to predict early relapse and median overall survival of low‐, intermediate‐ and high‐risk group according to the nomogram. (A) The decision curve analysis shows the clinical usefulness of the nomogram for early relapse. The *x*‐axis labels a continuum of potential thresholds probability for early relapse and the *y*‐axis labels the net benefit. The black dotted curve represents the prediction nomogram for ER. The red dotted curve represents the prediction of R‐ISS stage. The gray line assumes intervention for all and the thin black line represents intervention for none. (B) OS of each risk group by the ER prediction nomogram. Patients were divided into three groups under the score of the prediction nomogram: score > 120 as a high‐risk group, 60 < score < 120 as an intermediate‐risk group and score < 60 as a low‐risk group.

The decision curve analysis was used to compared the clinical efficacy of different prediction models. The x axis indicated a continuum of potential thresholds probability for ER and the y axis labeled the net benefit of the model. As showed in Figure 4A, the clinical utility of prediction nomogram for ER performs larger benefit across the range of ER risk than R‐ISS stage system (Figure 4A). According to the nomogram score, we divided patients into three groups: score > 120 as a high‐risk group, 60 < score < 120 as an intermediate‐risk group and score < 60 as a low‐risk group. The OS in three groups showed significant difference for OS (35.6 m, 95% CI: 18.6–59.6 vs. 62.4 m, 95% CI: 55.2–75.9 vs. 86.3 m, 95% CI: 72.8–103.3, *p* < 0.0001) (Figure 4B). In addition, the proportional hazard assumption was tested and the Cox model respects the proportional risk hypothesis (see Data S1).

### 3.8 Validation of the predictive accuracy in the validation cohort

The median OS was 50.6 months (16.2–80.4 months). The C‐index of the nomogram for predicting ER was 0.73 (95% CI, 0.64–0.81), and the calibration curve performed good concordant between predicted and actual 12‐month and 18‐month TTP probability (Figure 3C, D). The C‐index of the R‐ISS stage was 0.538 (95% CI: 0.46–0.62), which was significantly lower than that of the nomogram. The discrimination of the nomogram was better than that of the three other staging systems (C‐index: 0.73 vs. R‐ISS (0.54), ISS (0.55), and DS staging system (0.53)).

## 4 DISCUSSION

ASCT has emerged as the standard strategy in managing MM in patients less than 65 years old over the last decade.^19^ However, ER after ASCT remains a significant challenge owing to its poor prognosis and quality of life. It is crucial to predict whether a transplant‐eligible patient is likely to experience ER. Our analysis revealed that existing MM staging systems (the R‐ISS, ISS, and DS staging system) are not sufficient to significantly discriminate ER risk. None of the systems were explicitly developed for post‐ASCT ER prediction. We observed that the C‐indices were 0.59 (ISS stage), 0.52 (DS staging system), and 0.62 (R‐ISS) for ER prediction in our training cohort.

ASCT has been a cornerstone of MM therapy as its remarkable improvement of PFS and OS, especially for high‐risk MM.^20^ Under the diagram of ASCT consolidation /maintenance therapy, ER after ASCT remains an adverse event for survival. Except for double ASCT, strategies for the poor response after ASCT and ER are an unmet need. Studies showed consolidation therapy even after ASCT could improve response including CR and VGPR rates and PFS; however, a translation to improved OS has to be established.^21^, ^22^, ^23^, ^24^, ^25^ The EMN02/H093 reported that compared with no consolidation therapy, consolidation therapy with VRD prolonged PFS of 13 months, with no improvement in OS. However, the benefit of OS, particularly in the high‐risk group was just observed in patients who received intensification therapy of double ASCT.^26^ While the current daratumumab‐based first‐line trial did not show superiority of survival in high‐risk patients.^27^ In terms of MRD‐driven therapies, 3‐drug or 4‐drug combinations as consolidation after ASCT could help improve MRD‐negative rate, which may translate into longer survival. Several studies have examined high‐risk MM could benefit from posttransplant chimeric antigen receptor (CAR) T cell infusion as candidates for front‐line consolidation therapy.^28^, ^29^, ^30^ Current studies may prove to be controversial. Whether new generation of novel therapy could improve or overcome the adverse survival of high‐risk patients needs to be further investigated in the future clinical trial. Post‐ASCT consolidation/maintenance for therapy options in high‐risk and potential ER patients still needs to be optimized.

In our study cohort, we enrolled 420 patients who received novel agent‐involved induction therapy followed by ASCT at three centers in China. In this study, we developed a prognostic nomogram that had a significant association with ER. The prognostic model combines the response after ASCT and two clinical factors (high‐risk cytogenetics and LDH) in MM patients undergoing ASCT. Validation of the nomogram showed that it was effective at identifying patients at high risk of ER among transplant‐eligible MM patients. Our nomogram for ER provided better clinical usefulness assessed by decision curve analysis as well. The decision‐analytic measures focus on net benefit as key part for prediction models, which was developed to summarize the performance of the model in decision making.^31^ The difference between our predicted nomogram and R‐ISS stage mainly involves the addition of therapeutic response after ASCT, which may come up with the question that if consolidation paradigm after ASCT for inferior response patients could improve response and PFS.

Nomogram was more accurate and have advantages over conventional staging systems.^32^, ^33^ Thus, we proposed a prognostic nomogram for ER (<12 months) after ASCT for transplant‐eligible MM patients. The nomogram executed well in predicting ER, and the C‐index (0.75 for the training cohort and 0.73 for the validation cohort). The calibration curve showed good accuracy as well. As ER was an adverse factor for survival, we further divided the train cohort into low‐risk, intermediate‐risk, and high‐risk groups according to scores of the nomogram model. Kaplan–Meier analysis showed a significant difference in OS among those three groups (Figure 4B), which prove the effective clinical utility of the model.

To date, there has been no uniform conclusion regarding the prediction of ER. According to our study, R‐ISS stage III could also help predict ER, which was in agreement with the study of Sathish Gopalakrishnan et al.^13^ Many studies have suggested that age is an important predictive factor for ASCT outcome.^34^, ^35^ As we know, R‐ISS stage encompassed ISS stage and high‐risk cytogenetics. In our univariable analysis, there was no statistical significance of ISS stage. Therefore, high‐risk cytogenetics was selected to avoid confounding factor in R‐ISS stage system. Our study found that age was unrelated to ER after ASCT because patients underwent ASCT was physically young and fit. As to renal impairment, the incidence was 20.7% in the training cohort without severe renal impairment. Patients enrolled ASCT experience renal function improvement after novel drug‐containing induction treatment. In our analysis, response before ASCT had no significant difference. The reason may partly lay in that in real‐world practice, time for patients underwent ASCT was not consistent across different centers. We could not confirm whether patients achieve response platform and status of minimal residual disease before ASCT, which is also a potent limitation of this study.

Moreover, our prognostic nomogram model included a response less than VGPR as an independent predictor of ER after ASCT. The correlation of response and prognosis has been highlighted in previous studies.^9^, ^36^ The patients who did not achieve CR or VGPR were likely to relapse more quickly. The prognostic model reported by Chitrita Goswami et al.^37^ also highlights the importance of therapeutic response in multivariate analysis. Unlike the model in the present study, the model of Chitrita Goswami et al.^37^ included the response to induction therapy as an independent prognostic factor. Our study included the response after ASCT. Due to the sample paucity, interpretation of the response of different periods of disease should be validated in further studies.

In this study, ER patients accounted for a significantly higher percentage of high‐risk cytogenetics patients [del17p, *t* (14;16), or *t* (4;14)] at 41.9% compared with 25.6% in the non‐ER patients of the training cohort, which is consistent with previous reports.^8^, ^9^, ^38^, ^39^ This again highlights the importance of high‐risk cytogenetics in predicting outcomes. However, the mechanisms of how they create an aggressive course in cells are unknown.^40^ Keats et al.^41^ performed serial genomic tests and found that clones with high‐risk cytogenetics might survive during initiation treatment and become the dominant clone. The clonal evolution and heterogeneity of MM may need to be assessed in the ER population in future studies.

Our study showed that serum LDH > ULN was a strong ER indicator, consistent with previous studies.^42^, ^43^, ^44^, ^45^, ^46^ The importance of LDH should be emphasized. LDH is of prognostic value and parallels disease activity in advanced lymphoma patients. More emphasis should be placed on the significance of LDH in MM.

This study has limitations as well, because it was a retrospective and nonrandomized research. The following problems led to a narrow interpretation of our study. First, research^24^ has shown that negative minimal residual disease (MRD) might lead to a better outcome even in high‐risk and ER cases. MRD detection was not available in previous years, and the threshold differs in different centers, which led to the lack of analyses of MRD and ER. Second, the lack of data on conventional therapy treated patients, maintenance therapy and PFS2 therapy. Third, the correlation between pretransplant response was nonsignificant. Unfortunately, the analysis could not consider more pretransplant variables to predict ER before ASCT. At last, for the lack of an external database to further prove utility and validation of the model. As the prediction model was built in the context of Chinese population, hence its usefulness is limited. The model requires constant validation and updates based on the evolution of MM treatment, and even in people of different races. Further validation will be considered in another multicenter matched cohort or our single‐center cohort at different period. However, ASCT is still a vital process in improving outcomes, even for high‐risk patients. Our results should help strengthen posttransplant treatment strategies for high‐risk ER patients, such as prolonged consolidation treatment and proteasome‐IMiDs combined maintenance therapy.

In conclusion, ER after ASCT still appears to be a vital independent adverse prognostic factor even in the era of novel agents. As presented in this study, the nomogram objectively and accurately predicted ER and OS in transplant‐eligible MM patients undergoing novel agent treatment. High‐risk ER patients may need an updated post‐transplant strategy to achieve better outcomes. Additional researches are obligatory to further validate this model.

## AUTHOR CONTRIBUTIONS

**Huixing Zhou:** Conceptualization (lead); data curation (lead); formal analysis (lead); resources (equal); writing – original draft (lead); writing – review and editing (lead). **Yuan Jian:** Data curation (equal); funding acquisition (equal); resources (equal). **Juan Du:** Data curation (lead); formal analysis (equal). **Junru Liu:** Data curation (equal); methodology (equal); supervision (equal); validation (equal). **Zhiyao Zhang:** Data curation (equal). **Guangzhong Yang:** Data curation (equal); software (equal). **Guorong Wang:** Data curation (equal). **Ying Tian:** Data curation (equal); formal analysis (equal). **Yanchen Li:** Conceptualization (equal); data curation (equal). **Yin Wu:** Conceptualization (equal); data curation (equal); supervision (equal). **Wenming Chen:** Conceptualization (equal); resources (equal); supervision (lead); writing – review and editing (equal). **Weijun Fu:** Conceptualization (equal); data curation (equal); supervision (equal); writing – review and editing (equal). **Juan Li:** Conceptualization (equal); data curation (equal); investigation (equal); validation (equal); writing – review and editing (equal). **Wen Gao:** Conceptualization (equal); data curation (equal); validation (equal); writing – original draft (equal); writing – review and editing (equal).

## CONFLICTS OF INTEREST

The authors have no conflicts of interest.

## ETHICAL APPROVAL

The Ethics Committee of Beijing Chao‐Yang Hospital approved this study following the Declaration of Helsinki.

## Supporting information


Data S1

## Data Availability

No additional data are available, as no new data were created or analyzed in this study. The data are not publicly available due to restrictions that could be available on the privacy of research participants.
