# Supplementary material for: Prognostic nomogram for multiple myeloma early relapse after autologous stem cell transplant in the novel agent era
Source: Cancer Med. 2023 Apr 6;12(8):9085–96. doi: 10.1002/cam4.5630 (PMC10166899; doi:10.1002/cam4.5630)

**Testing the proportional hazard assumptions**

>ERmod<-coxph(Srv~response_after+HIGH+LHD,data = train)

> temp <- cox.zph(ERmod)

> print(temp) # display the results

chisq df p

response_after 8.59e-01 1 0.35

HIGH 1.65e+00 1 0.20

LHD 4.18e-05 1 0.99

GLOBAL 2.26e+00 3 0.52

> plot(temp) # plot curves


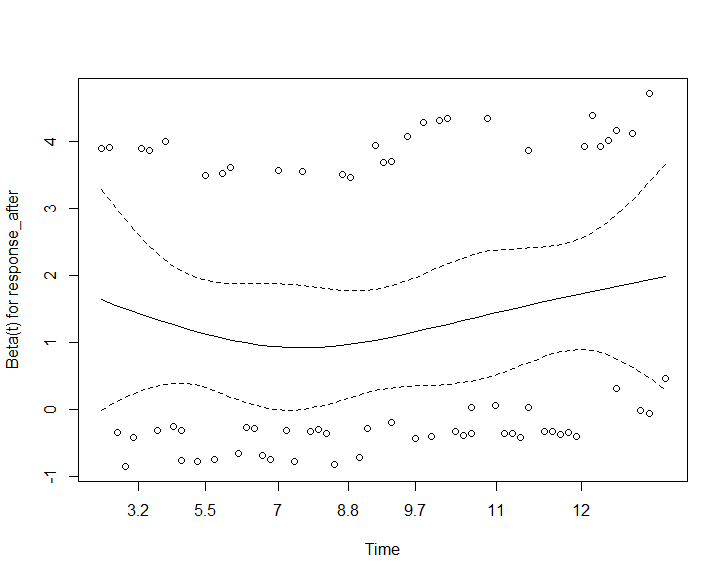


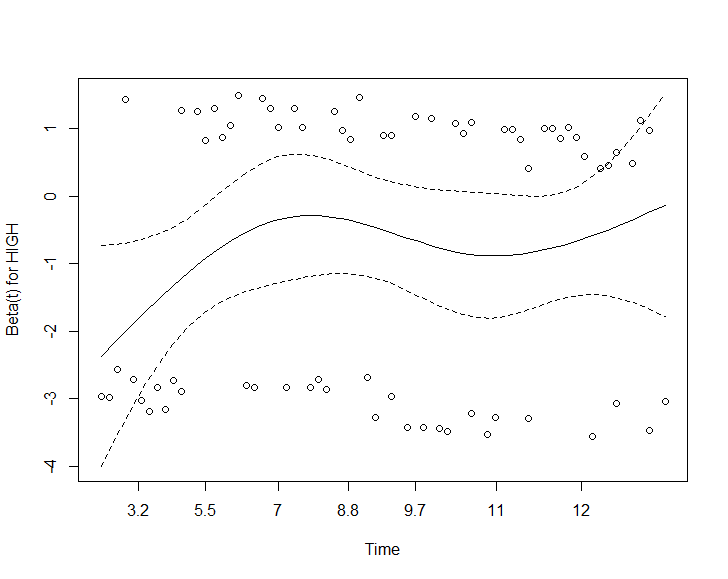


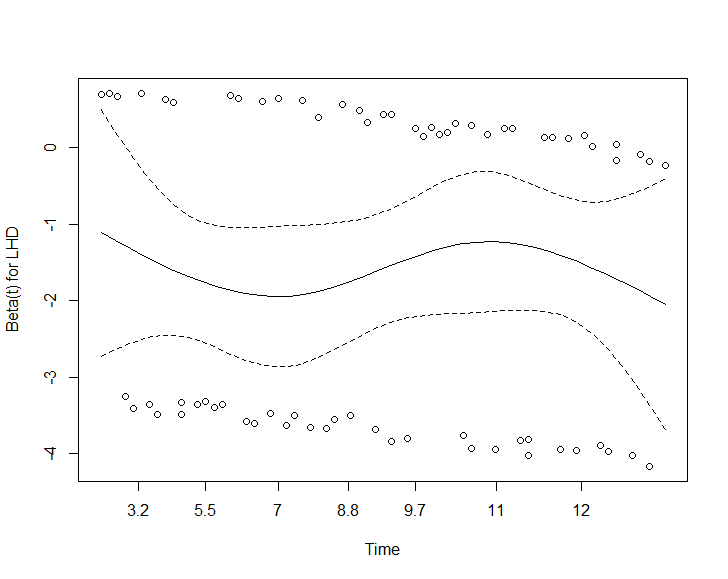

Supplement: Supplementary file 1 — Data S1 [file CAM4-12-9085-s001.docx]
